# Value Benefit Analysis Software and Its Application in Bolu-Lake Abant Natural Park

**DOI:** 10.3390/s8095745

**Published:** 2008-09-17

**Authors:** Alper Aytekin, Omer Lutfu Corbaci

**Affiliations:** Bartin University, Forestry of Faculty, 74100, Bartin, Turkey; E-mail: omerlutfucorbaci@hotmail.com

**Keywords:** Value Benefit Analysis (VBA), Computer Software, Bolu-Lake Abant Natural Park (BLANP)

## Abstract

Value benefit analysis (VBA) is a psychometric instrument for finding the best compromise in forestry multiple-use planning, when the multiple objectives cannot be expressed in the same physical or monetary unit. It insures a systematic assessment of the consequences of proposed alternatives and thoroughly documents the decision process. The method leads to a ranking of alternatives based upon weighting of the objectives and evaluation of the contribution of each alternative to these objectives. The use of the method is illustrated with hypothetical data about Bolu-Lake Abant Natural Park (BLANP). In this study, in addition, computer software controlling the confidence was created. This software puts into practice the method proposed by Churchman and Ackoff, and determines the significance of the alternatives quickly and accurately.

## 1. Introduction

Society's needs for forest products and services have been changing over time, depending on the development process. In planning, the various elements are measured and evaluated with assigned minimum and maximum values and indirectly for intangible assets. Furthermore, instead of being single-purposed, goal determinations that can match the desires and trends of a society are preferred.

Through recreation and tourism planning, which have increasing importance nowadays, many methods related to a nation's preferences with regards to free time use have been employed in the determination of the best uses for various regions [1, 2]. Nowadays, free time activities and different desires concerning open spaces are being examined and evaluated. “Value Benefit Analysis (VBA)”, which is based on subjective value understanding, can be considered a systematic evaluation tool in this respect. In other words, the subjective value of an area is a positive value which meets a need [3]. VBA is a method that provides a systematic evaluation of options [4, 5]. It is an evaluation of a system composed of a desired number of options (n>2) and suitable criteria that meets some stated needs. Therefore, the total value is a common result that considers and includes all criteria of an option [6].

The ratings given to the specified data measure the potential to meet the needs of visitors and include all criteria on open space use. These ratings are between 0 and 5 (0.0-unsuitable, 2.5-suitable, 5.0-very suitable, etc.).

One of the key targets of forestry is to have multiple values. In other words, it attempts to utilize the products and services that forests provide together and in the same area. The importance of recreational activities, rest and the recreation concept itself has been increasing with leisure time, which is an indispensable part of individual lifestyles. The determination of the importance level of multiple value options of the area is the main step of specifying the purpose. The reason why the Bolu-Lake Abant Natural Park (BLANP) was selected a case study is that it has been divided into three options, identified as “picnic areas”, “forest lands” and “lake and its environs”. An evaluation and the flow sheet of analysis of related options and criteria have been explained with the aid of figures and tables. In addition, the software developed has been introduced by screen captures. The software has been written n Delphi^®^ 2006 programming language. The minimum requirements for the software are: Pentium (or above) processor, 512 MB of memory and Windows XP (or higher) operating system. The software is Windows Vista compatible and the data to be used can be transformed into various formats.

## 2. Materials and Methods

### 2.1. Material

Lake Abant and its environs, located 34 km to the south east of Bolu, is an avalanche lake which has an altitude of 1,358 m and an total area of 125 ha, including the surrounding natural park, covered with pine and fir trees. The maximum depth is 18 m. The lake is fed by a few spring waters, partially continuous rivers and snow and rain waters in particular [7].

The BLANP is a recreational area for relaxation and entertainment with its wildlife, flora and panoramas and composed of a natural lake and surrounding forests. In 1988, 1150 ha of the area was declared a natural park by 2^nd^ paragraph of 3^rd^ Article of No: 2873 National Parks Law. In 1991, there was a border revision at the park that added 26 ha more to the 1988 area [8].

National parks are parts of nature that usually have superior visual richness and wildlife features. The important differences that set natural parks apart from national parks are that they are open to recreational uses, with natural richness and beauties that may not be found elsewhere at a national level [9]. Natural parks are parts of nature having only protected, relaxation and tourism areas and rare natural values [7].

**Figure 1.**
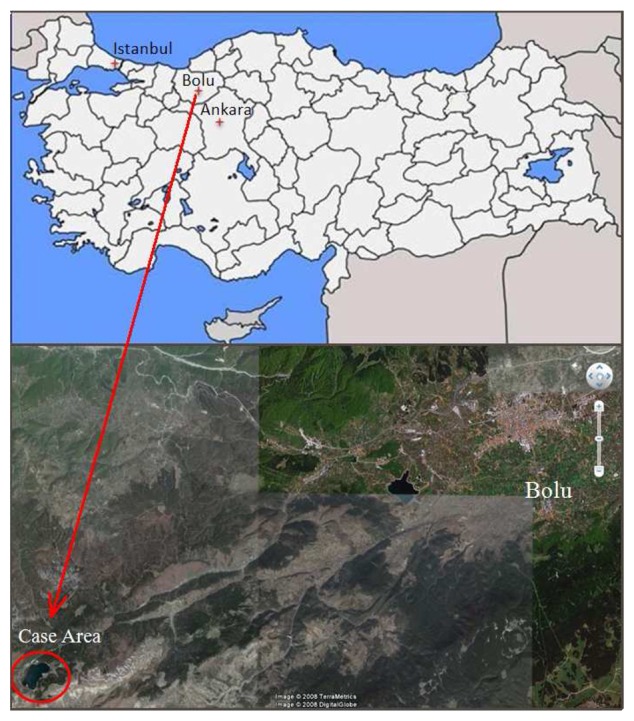
Geographical position of case area.

The lake, which is of natural and eutrophic-character, lies parallel to the Black Sea coast of the Western Black Sea geographical region and is located on the secondary mountain ranges of Turkey. It is 240 km away from Istanbul, 210 km away from Ankara and 22 km away from highway E5 (Figure 1). The Abant region is included in Europe-Siberia phytogeographic region. The park is surrounded by mountains having elevations of up to 1,700 m [8].

The data obtained from the Bolu Meteorological Station have been used since there is no meteorological station nearby the park. According to the data, the region is cold and rainy in winter and has a characteristic transition between Mediterranean and Oceanic climate. Due to its geographical position, the region is totally closed to Southern winds but open to Northern winds, causing high moisture. Total annual rainfall is 545.6 mm, while it is 223.5 mm during the vegetation period. Average annual temperature has been calculated at 7.1 C° and the park is 600 m higher than Bolu. The hottest and the coldest days were measured at 20 C° in August and −9 C° in February [10].

The environs of the lake have a rich flora. The most significant forest trees are *Pinus nigra* Arnold (Black pine), *Pinus sylvestris* L. (Scotch pine), *Abies nordmanniana* (Stev.), *Abies bornmülleriana* (Uludağ Fir), *Populus tremula* L. (Aspen), *Fagus orientalis* Lipsky., (Eastern Beech), *Acer sp.* (Maple) and *Salix sp.* (Willow). The most abundant shrubs are *Crataegus sp.* (Hawthorn), *Tamarix sp.* (Tamarisk), *Corylus sp.* (Hazel), *Ilex sp.* (Holly), *Juniperus sp.* (Juniper) and *Vaccinium sp.*) [8].

**Figure 2.**
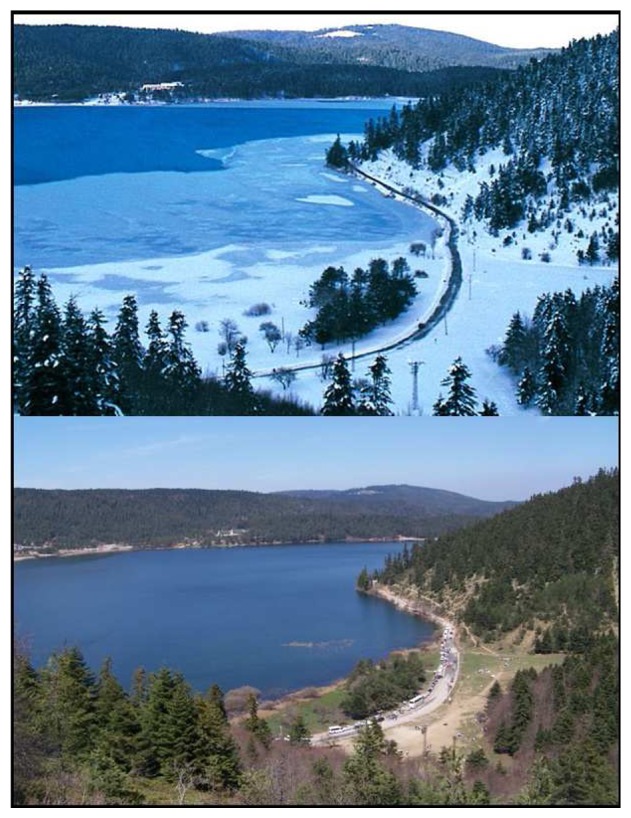
A vista point in BLANP in winter and summer (www.hasseyahatdergisi.com).

The park contains a small city which people use in both summer and winter (Figure 2) for their holidays, with lots of hotels and restaurants, cafeterias, a post office, a disco, forest lands and picnic areas. However, the stationary population is very low in winter.

### 2.2. Methods

#### 2.2.1. VBA of open space areas

The definition of the purpose a system must meet is the first step required in the evaluation of open spaces. According to Krahl the key targets below have to be considered in the introduction of open spaces [5]:

- Open spaces should meet the recreational needs of people in the best way.
- Open spaces should broadly fulfill various protection and appearance functions.
- Separate area options should provide a movement and play ground for free activities and highly meet different desires of each individual.

**Figure 3.**
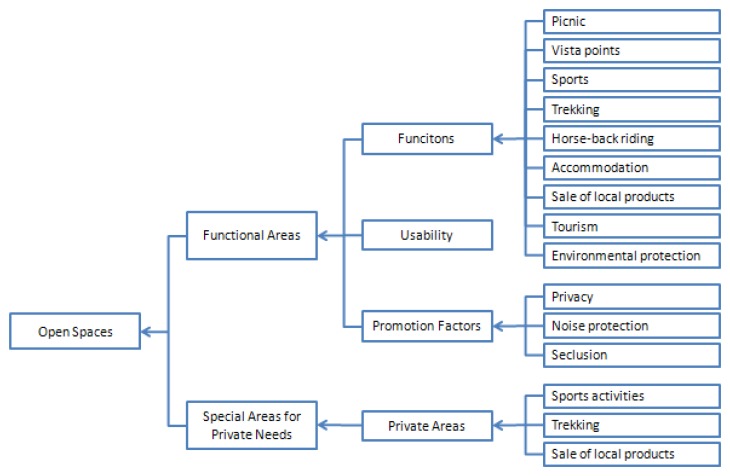
The goal hierarchy for VBA of the BLANP opens space.

These targets should be ordered as a goal hierarchy. The goal hierarchy of the park which has been developed systematically is given in Figure 3.

#### 2.2.2. VBA Model

**Figure 4.**
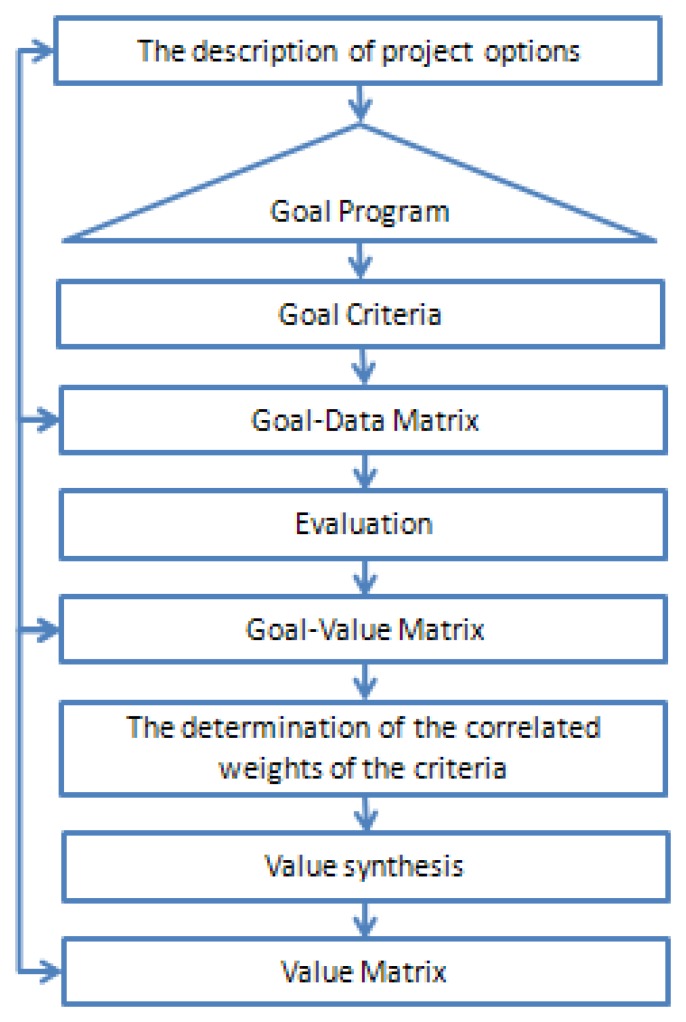
Flow sheet of VBA.

The flow process of VBA is based on the principle that the options are evaluated directly (without an intermediary). Figure 4 shows the operations included in this process.

##### 2.2.2.1. The description of project options

Application of a theoretical formula in this stage of work is not desired. Options are partially preconcerted according to goal condition and by taking priorities into account. Three options determined for the park are: picnic areas, forest lands and lake with its environs. The level of importance of these options can be established according to the desired results.

##### 2.2.2.2. Developing of the goal program

A goal program is developed as the second step of solution. In other words, all of the goals are sought and found as appropriate to decide and then the complete evaluation is listed systematically to form a base for the goal system arranged hierarchically. These are:

- Formation of a mixed hierarchy (listing),
- Gradual arrangement and completion of goals for a goal system arranged as a hierarchy,
- Formalization of goals by parceling or transformation of sub-goals to goal criteria,
- Sectioning of goals, showing them part by part including criteria applied,
- While goals are determined as a list, they can be specified as the combination of principle goals and directly evaluated as a group.

Goal criteria should require “freedom of limited value”. Therefore, related participation ratio of total benefit value of each criterion will easily be specified [4].

##### 2.2.2.3. Formation of the goal-data matrix

In the third step of the solution the options are described in detail by the formation of a goal-data matrix. The criteria are measured according to their properties or effects (km, %, piece, etc.) and the compatibility with the related goal is then determined. In the drawing up of goal-data matrix, each goal is directly evaluated for each option and the solution is reached by showing it in goal-data matrix (Table 1). Separate elements of goal-data matrix E_ij_ is denoted weightlessly either by text or numbers according to goal values (matrix of values correlated weightlessly).

##### 2.2.2.4. Formation of goal-value matrix

In the fourth step of solution, the criteria of options are mutually compared and classified according to order of priority (Table 2). In other words, several of the elements are compared with respect to their common properties. In the formation of goal-value matrix, the options are assessed gradually according to their values. This feature requires the arrangement of the values and the options which can be determined according to separate criteria. As seen in goal-data matrix, effective elements can usually be determined neither by practical markers nor in terms of quantity such as m^2^ or kg. Therefore, the use of perception (intellectual evaluation) or “optional” scales are required. Equation 1 and 2 are used to determine W_ij_ and weights.

(1) $$W_{ij}=\frac{E_{ij}}{N_{i}}\times 100$$

(2) $$G_{j}=\frac{\sum_{i=1}^{n}W_{ij}}{\sum_{i=1}^{n}\sum_{j=1}^{x}W_{ij}}\times 100$$

where:

*n* = the number of options
*x* = the number of criteria in each criterion area

##### 2.2.2.5. The determination of the correlated weights of the criteria

Separate properties are taken into account by assigning different weights in multi-staged goal systems. Therefore, the correlated weights of the criteria are determined in the next step. The method proposed by Churchman and Ackoff [12] has been utilized for comparison with respect to priority. The goal criteria are listed in order according to correlated importance so that the most important and the least important criteria are located at the top and the bottom.

Separate criteria for an open space to be designed include significant differences in terms of their incompatibilities. “Weight elements” have been developed to determine separate arrangement values. This determination process is mostly affected by the goal system. Therefore, in weight distribution, the determinations about top criterion area should be performed first. In order to let mutual control and equalization other known information is included in the process. “Staged comparison method” has been employed in mutual control and equalization process.

##### 2.2.2.6. Value synthesis

All required values have been obtained to determine the values of the open space in finalizing weight determination. First, real participation rate and correlated benefit value of each criterion belonging to the value of open space should be specified. In other words, established criteria values (E_ij_) are multiplied by related weights (G_j_) and correlated benefit values (T_ij_) are obtained (3).

(3) $$T_{ij}=G_{i}\times E_{ij}$$

Then, value synthesis is finalized by finding the value of that open space (F_j_) calculated by summing up the correlated benefit values (T_ij_) (4).

(4) $$F_{j}=\sum_{i-1}^{n}G_{i}\times E_{ij}$$

## 3. Results and Discussion

### 3.1. The formation of goal-data matrix

**Figure 5.**
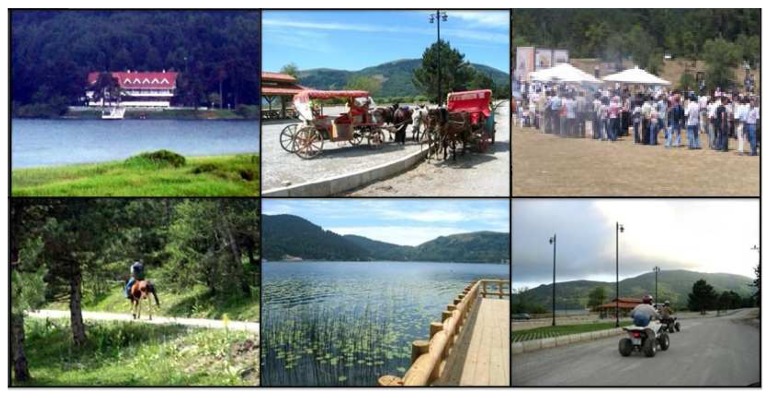
The functioning opportunities of criteria in open space (www.pedalsesi.com).

While the functioning opportunities of criteria in open space are determined by writing and numbers between 0 and 5, their potential in meeting visitors' needs are explained (Figure 5 and Table 3). As a result of Goal-Data Matrix evaluation of the park, in picnic criteria, “picnic” has been found to be the most important element with 4.4 points, having many landscape opportunities and adequate availability of equipment (picnic tables, taps, wash basins, barbeques, etc.), leaving appropriate places for children's playgrounds, volleyball and basketball fields and the like.

Within the forest lands criteria, “vista points”, with 4.5 points, has been found to be the most important element. The study area exhibits many different areas of scenic beauty in all four seasons. It is possible to see the harmony between the lake and the plants. Trees having foliation like *Fagus orientalis* Lipsky. (Eastern beech) and *Acer sp.* (Maple) and evergreen trees like *Pinus nigra* Arnold. (Black pine), *Pinus sylvestris* L. (Scots pine) and *Abies bornmülleriana* (Uludağ fir) provide beautiful scenary in autumn.

As a result of Goal-Data Matrix evaluation of open space of the park, noise control has been determined as the lowest component in picnic criteria with 1.2 points. Intense, patchy and uncontrolled use of the area causes noise control to be the lowest component.

Within the forest lands criteria trade of regional products has been found the lowest element with 1.2 points. The products produced by the locals (tarhana, homemade macaroni, pine honey and the likes) are sold along the road around the lake.

The protection of nature has been determined as the lowest component within the lake and its environs criteria by 1.2 points. Current activities on coast of the lake cause negative effects on the ecosystem (water pollution, effects on flora, fauna, etc.).

### 3.2. The formation of Goal-Value matrix

The goal criteria of the options have been mutually compared and partitioned by order of priority (Table 4). The functioning opportunities of picnic areas, forest lands and lake and its surrounding options have been calculated at 71, 63 and 66 %, their area values have been calculated at 16, 20 and 16 % and incentives have been specified as 14, 17 and 18 %. The sum of the functioning opportunities and area values of each option gives 100 %.

### 3.3. The determination of correlated weights of the criteria

To determine the weight criteria values the options in the goal-value matrix are separately summed and written in column G_j_ in Table 5. “Transformation coefficients” are obtained by dividing the sum of those values by 100. Percentages of shared weights of the criteria are found by multiplying G_i_ values of the criteria of three options by obtained coefficients (i.j). For example, 12.8, which is the total of all three options of picnic criteria is multiplied by the transformation coefficient 1.12 to give 14.

### 3.4. Value synthesis

To complete the value synthesis T_11_ is found to be 4.93 (1.12×4.4) for the picnic criterion of the picnic option. Within the three options of our example lake and its environs occupies the top importance by F_3_=111.20. Arrangement of correlated benefit values and flow of value synthesis are shown in Table 6.

As a result of the column sum of correlated benefit values (T_ij_), the priority of importance for Bolu-Lake Abant Natural Park has been specified according to VBA as below:
1.The lake and its environs100 %F_3_=111.202.Forest lands95%F_2_=105.503.Picnic areas75%F_1_=83.30

### 3.5. The utilization of software

The software has been developed to obtain the results after applying the procedure mentioned above for criteria and determined values. After running the software, the user should follow this processes: the “New” button is clicked if the data are introduced for the first time or “Open” button is clicked to open formerly saved data.

**Figure 6.**
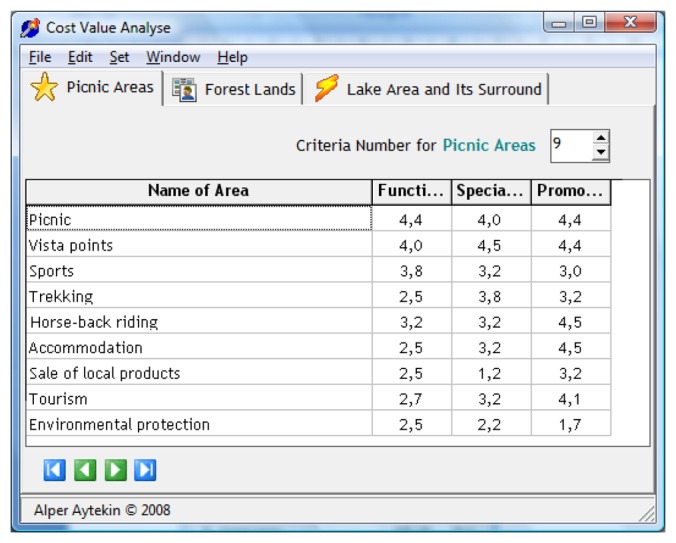
Data entrance section of the software.

**Figure 7.**
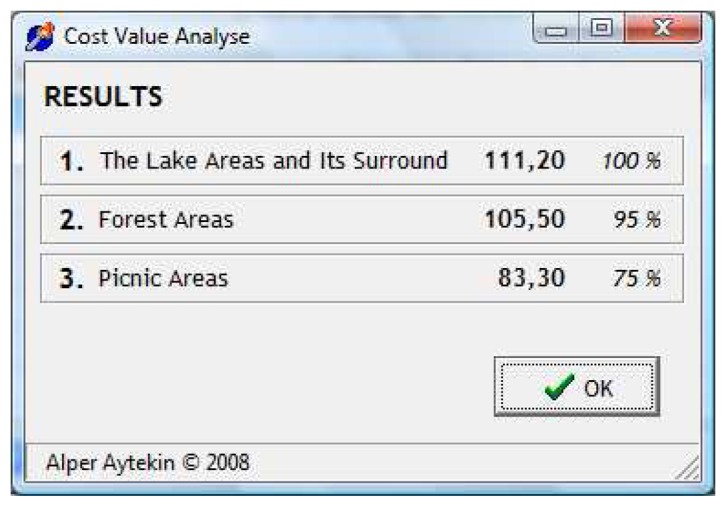
Visualization of the result table by the software.

In the new data section; after entering the number of the options (i), the names of the options (A_1_, A_2_,…,A_i_), the number of the criterion area (x), the names of these areas (KB_1_, KB_2_,…,KB_x_), the number of the criteria to be evaluated for each criterion area (j) and their names (K_1_, K_2_,…,K_j_), data entrance section (Figure 6) comes to the screen where data are entered (E_11_, E_12_,,,,,E_21_, E_22_,…, E_ij_). Once the data entrance process is completed these values are calculated and visualized by pressing F5 (Figure 7).

## 4. Conclusions

VBA is the evaluation of a system composed of suitable criteria that meet a desired possible number of options and needs (n>2). The method proposed by Churchman and Ackoff [12] has been utilized in VBA of the BLANP. The priority of importance of the options (picnic areas, forest lands and lake and its environs) and criteria used in the array of goal-value matrix has been determined for the park.

The landscape value of BLANP is quite high. It is an area where various recreational activities can be carried out. When landscape value, climate value, accessibility, recreative values and negative effects are taken into account, it can be concluded that the park is a region where people can spend their spare time for short or long-term stays. Furthermore, the region has a high potential for future recreation activities.

The plan is to make the BLANP a premium tourism area. In order to improve recreation activities, several recommendations are made, such as protecting the ecological balance, pollution prevention, building a ski-center, a bicycle road, a railway system, landscape terraces and the road links between them, and prevention of visual pollution by supplying an underground electricity line.

According to the methods developed by Altan [13] and Gulez [14], while actual recreation potential is determined subjectively, the priority of importance of the options and the criteria can objectively be determined by the software developed on the basis of the method proposed by Churchman and Ackoff [12]. The landscape value of an area can be determined precisely in a short time by means of the software.

## Figures and Tables

**Table 1.** Linear notation of a goal-data matrix [11].

**Criteria Areas**		**KB_1_**				**KB_2_**			**KB_3_**	

**Alternatives**	**Criteria**	K_1_	K_2_	K_3_	K_4_	K_5_	K_6_	K_7_	K_j_	K_m_

	A_1_	E_11_	E_12_	E13	E14	…	…	…	E_1j_	E_1m_
	A_2_	E_21_	E_22_	…	…	…	…	…	E_2j_	…
	A_3_	E_31_	E_32_	…	…	…	…	…	E_3j_	…
	…	…	…	…	…	…	…	…	…	…
	…									
	…									
	A_n_	E_i1_	…	…	…	…	…	…	E_ij_	E_nm_

**Table 2.** Linear notation of a goal-value matrix [11].

**Criteria Areas**		**KB_1_**					**KB_2_**			
				
**Criteria**		K_1_	K_2_	K_3_	K_4_	Σ	K_5_	K_6_	Σ	Σ

**Weight %**		G_1_	G_2_	G_3_	G_4_		G_5_	G_6_		100 %

**Alternatives**	A_1_	W_11_	W_12_	W_13_	W_14_		…	…	…	N_1_
	A_2_	W_21_	W_22_	…	…		…	…	…	N_2_
	A_3_	W_31_	W_32_	…	…		…	…	…	N_3_
	…	…	…	…	…		…	…	…	…
	…									
	…									
	A_n_	W_nl_	W_n2_	…	…		W_n5_	W_n6_	…	N_n_

										N_total_

**Table 3.** Summary for goal-value matrix of the BLANP open space.

		**Alternatives**		
**Criteria**	**Areas of Criteria KBx**	**Picnic areas**	**Forest lands**	**Lake area**
**Functions**	Picnic	4.4	4.0	4.4
	Vista points	4.0	4.5	4.4
	Sports	3.8	3.2	3.0
	Trekking	2.5	3.8	3.2
	Horse-back riding	3.2	3.2	4.5
	Accommodation	2.5	3.2	4.5
	Sale of local products	2.5	1.2	3.2
	Tourism	2.7	3.2	4.1
	Environmental protection	2.5	2.2	1.7
**Special Needs**	Privacy	3.8	3.2	4.0
	Noise control	1.2	2.5	1.3
	Seclusion	1.3	3.2	2.5
**Promoting Factors**	Sports activities	1.7	2.5	3.2
	Trekking	2.5	4.0	2.5
	Sale of local products	1.2	1.2	3.2

**Table 4.** Schematic representation of goal-value matrix of the BLANP opens space.

			**Alternatives (%)**		

**Criteria**	**Areas of Criteria**	**Weight %**	Picnic areas	Forest lands	Lake area

**Functions**	Picnic	14	11.0	9.0	9.0
	Vista points	14	10.0	10.0	9.0
	Sports	11	10.0	7.0	6.0
	Trekking	11	6.0	8.0	6.0
	Horse-back riding	11	6.0	7.0	9.0
	Accommodation	12	8.0	7.0	9.0
	Sale of local products	8	6.0	3.0	6.0
	Tourism	11	7.0	7.0	8.0
	Environmental protection	8	6.0	5.0	3.0

	**Total**	**100**	**71.0**	**63.0**	**66.0**

**Special Needs**	Privacy	48	10.0	7.0	8.0
	Noise protection	22	3.0	6.0	3.0
	Seclusion	30	3.0	7.0	5.0

	**Total**	**100**	**16.0**	**20.0**	**16.0**

**Promoting Factors**	Sports activities	34	4.0	6.0	6.0
	Trekking	41	6.0	9.0	5.0
	Sale of local products	25	3.0	3.0	6.0

	**Total**	**100**	**14.0**	**17.0**	**18.0**

**Table 5.** Weighted explanation of the criteria.

**Step**	**Number**		**Weight of the criteria (%)**	**G_i_**	
	
**i**	**j**	**Short Notes**	**Shared (i,j)**	**%**	

1	1	Functions	67	89.6	Total 134.6
	2	Special Needs	17	23.0	
	3	Promoting Factors	16	22.0	

2	1	Picnic	14	12.8	Total 89.6
	2	Vista points	14	12.9	
	3	Sports	11	10.0	
	4	Trekking	11	9.5	
	5	Horse-back riding	11	10.2	
	6	Accommodation	12	10.9	
	7	Sale of local products	8	6.9	
	8	Tourism	11	10.0	
	9	Environmental protection	8	6.4	
				
	10	Privacy	48	11.0	Total 23.0
	11	Noise protection	22	5.0	
	12	Seclusion	30	7.0	
				
	13	Promoting sports	34	7.4	Total 22.0
	14	Promoting games	41	9.0	
	15	Promoting recreation	25	5.6	

**Table 6.** Calculation scheme for VBA of open spaces.

**Areas of Criteria KB_X_**			**Alternatives**					
			Picnic areas		Forest lands		Lake area	
**Criteria**		**G_i_**	**E_i_**	**T_i_**	**E_i_**	**T_i_**	**E_i_**	**T_i_**
	Picnic	1.12	4.4	4.93	4.0	4.48	4.4	4.93
	Vista points	1.12	4.0	4.48	4.5	5.04	4.4	4.93
	Sports	1.12	3.8	4.26	3.2	3.58	3.0	3.36
	Trekking	1.12	2.5	2.80	3.8	4.26	3.2	3.58
	Horse-back riding	1.12	3.2	3.58	3.2	3.58	4.5	5.04
	Accommodation	1.12	2.5	2.80	3.2	3.58	4.5	5.04
	Sale of local products	1.12	2.5	2.80	1.2	1.34	3.2	3.58
	Tourism	1.12	2.7	3.02	3.2	3.58	4.1	4.59
	Environmental protection	1.12	2.5	2.80	2.2	2.46	1.7	1.90
	Privacy	4.35	3.8	16.53	3.2	13.92	4.0	17.40
	Noise protection	4.35	1.2	5.22	2.5	10.88	1.3	5.66
	Seclusion	4.35	1.3	5.66	3.2	13.92	2.5	10.88
	Sports activities	4.55	1.7	7.74	2.5	11.38	3.2	14.56
	Trekking	4.55	2.5	11.38	4.0	18.20	2.5	11.38
	Sale of local products	4.55	1.2	5.46	1.2	5.46	3.2	14.56
	**Total**		39.8	83.30	45.1	105.50	49.7	111.20
